# Editorial: Cell biology of brain development and evolution

**DOI:** 10.3389/fcell.2023.1147510

**Published:** 2023-02-07

**Authors:** Felipe Mora‐Bermúdez, Giuseppe Testa, Elena Taverna

**Affiliations:** ^1^ Max Planck for Cell Biology and Genetics, Dresden, Germany; ^2^ Human Technopole, Viale Rita Levi-Montalcini 1, 20157, Milan, Italy; ^3^ Department of Oncology and Hemato-Oncology, University of Milan, Via Santa Sofia 9, 20122, Milan, Italy

**Keywords:** brain evo-devo, stem cells, cell biology, brain organoids, astrocytes, vasculature, genomics, induced pluripotent stem cell (iPSC)

## Introduction

Since the early days of neurobiological research, embryological, anatomical and histological approaches have led the way in giving us fundamental information about how the nervous system evolves and develops, both in health and disease. Rather than being replaced, such classical approaches have been constantly updated and diversified: from the most basic preparation steps integrating advances in precision sectioning and tissue clearing, to observation and quantitation benefitting from the latest multi-photon, light sheet, correlative, and serial block-face electron microscopy advances, and complemented with automated tissue manipulation and image processing tools. In parallel, transformative advances in molecular biology methods for control and manipulation, all the way up to optogenetics, developmental optogenetics and gene-editing, have been seamlessly integrated to grow a powerful toolkit of innovative models, *in vitro*, *in vivo* and *ex vivo* alike, to probe brain development and evolution (Shinmyo et al.; Vaid and Huttner). Among the latter, the rise of 3D organoid cultures has been providing the invaluable capacity to bridge between 2D cell cultures and native tissues and organs, opening up spatiotemporal neural trajectories to unprecedented experimental tractability (Kaluthantrige Don and Kalebic). “Omics” techniques, including whole genome, epigenome, transcriptome and proteome, are also contributing to give us a more global and detailed view of species-specific differences, as well as of the transient vs. permanent states that each cell can adopt during development (Buisan et al.).

## Cell biology of neural stem and progenitor cells

The dawn of this new millennium has seen a major leap in our knowledge on the identity of mammalian neural stem and progenitor cells (NSPCs) and the mechanisms through which they ultimately generate all the neural lineages in the brain. Vaid and Huttner provide in this Research Topic a broad review of the cell biological features of neocortical NSPCs (ncNSPCs) and how they ultimately generate neurons by going from fully epithelial to partially epithelial to fully differentiated nerve cells. The analysis of these components has permitted to identify and characterise the major ncNSPC types, in both apical and basal zones of the developing cortical wall. In the past, knowledge about the diversity of NSPC types was intrinsically hampered by the lack of diversity of model systems. Therefore, an important focus is given here to the species-specific differences that have progressively been uncovered thanks to comparisons of the traditional cell-based and rodent models to more recent models appropriate for larger and more complex brains, such as those of gyrencephalic species. From their interphase morphology to their cell division behaviour, the mechanistic dissection of these differences will be crucial in the efforts to nail down the nexus between structure and function in the brains of different species.

The coordination between stem cells proliferation and differentiation is achieved by balancing different types of divisions, such as symmetric proliferative vs. asymmetric self-renewing vs. asymmetric neurogenic division. Casas Gimeno and Paridaen review the current status of research about division symmetry in the developing brain, and highlight the intrinsic dichotomy that the term entails: indeed, the term symmetry refers both to the fate symmetry/asymmetry, and to the cell biological basis of it. Apical radial glia (aRGs) offer the paradigm for the establishment of asymmetry, because of their extreme epithelial architecture, with a very small apical plasma membrane, bearing a primary cilium protruding into the ventricle, a tightly regulated angle of mitosis and an extremely elongated basolateral plasma membrane. Interestingly, all these features have been implicated as regulators of asymmetric division and fate, begging the question of how, if all these different sources of asymmetry are potentially at work, the information they carry can be functionally integrated at the single cell level. A possible explanation emerging from recent studies suggests that these mechanisms are strongly cell type-, region- and stage-specific, adding time and space to the complexity of neural progenitor cell biology.

### Cellular players

Recent work has uncovered an unexpected diversity of neural stem and progenitor cells in development and evolution. However, as the studies deepen, it is becoming obvious that an understanding of brain in ontogeny and phylogeny requires extending of our gaze beyond the neural lineage to incorporate systems and cell types that are increasingly claiming central stage: the astrocytes and the vascular system.

Falcone elucidates the present status of research on the evolution of astrocytes, reviewing what is known from invertebrates to primates, discussing a clear trend towards increased complexity in the structure and function of astrocytes. Apart from quantitative differences, there are also qualitative difference in primates, with a specific cell type identified as being primate specific. These findings highlight the relevance of looking at all cell types when studying brain evolution and should invite us to consider cellular complexity and diversity when studying human neurodevelopmental disorders as well. In addition, it is for us intriguing to note that the increase in astrocyte’s structural complexity mirrors an increase in morphological complexity also of bRGs during evolution, warranting a focused effort to identify the underlying genetic drivers of this increased complexity in both cell types and probe the extent of their overlap.

*The vascular system* provides tissues with oxygenation and metabolic substrates, yet while the negative consequences of hypoxia during brain development are well established, the long-term consequences of oxygen excess, remain less clear, despite its potential therapeutic use to compensate for insufficient brain growth caused by genetic and environmental factors. Markert and Storch in this Research Topic use their established *in vivo* hyperoxygenation model system to contribute a necessary analysis of the long-term effects of hyperoxygenation throughout mammalian brain neurogenesis. They confirm that more neurons can be generated by hyperoxygenation during mid-neurogenesis, specifically more Layer 5 (L5) neurons and associated synaptic markers. These effects are, however, shown to be non-permanent, and the L5 neuron and synaptic marker numbers come back to wt levels shortly after birth. Interestingly, their results also suggest that microglia, rather than apoptosis, could be responsible for selectively eliminating the excess number of neurons. It will be interesting to further explore the duration of the effects of hyperoxygenation in different models of brain disorders or injury.

Vogenstahl et al. illustrate the role of the vasculature in developmental neurogenesis and the change in paradigm we have been witnessing in the last decades, with the vasculature moved from being recognized for its well-known role of nutrient supply, to a more regulatory and instructive role both in hindbrain and in forebrain. Specifically, two processes have emerged as crucially dependent on vasculature: neurogenesis and neuronal migration. The vasculature provides a niche for basal progenitor division and signaling, with the basal process of RGs also physically associated with vessels. It will be relevant to dissect the molecular mechanisms and outcomes of this intimate interplay to better define the nature of the basal progenitor/vasculature niche, including also a mechanobiological account of the vasculature and bloodstream as pressure carrier.

## Modelling different brain architectures

When analyzing differences between the brains of different clades, few differences are as conspicuous as the structure of the outer surface of the cerebral cortex. In gyrencephalic mammals, the appearance of folds in the outer surface of the cortex is thought to have arisen from an interplay between the larger lateral expansion of the basal zones of the developing cortical wall -as opposed to the more constrained apical ventricular zone-, and the spatial limits imposed by the size and shape of the skull. In these Research Topic, Shinmyo et al. and colleagues provide a timely review of efforts to elucidate the functions of these folds as well as the mechanisms responsible for their formation. Focalized attention is given to the use of appropriate model organisms to study different brain architectures. Whereas the mouse is an appropriate model for the study of the mammalian lissencephalic architecture, it falls short when studying the gyrencephalic architecture. The ferret has therefore emerged as a relevant alternative to probe the intricacies of cortical brain folding that could be potentially extrapolated more meaningfully to the human setting. Being a relatively small domesticated mammal, as well as having a long history of use in biomedical research has made ferrets an attractive animal model for studying brain development and evolution. Shinmyo et al. and colleagues also shed light on an often overlooked but important aspect of brain folding, namely the development of the cortical fiber layers.

## The iPSCs (r)evolution

iPSCs technology has revolutionized the way we study the human condition at the cellular and molecular level, allowing us to study key aspects of human brain development and neuronal maturation as they unfold *in vitro*.

### iPSCs-derived neurons

A glimpse into the human condition is given by the possibility to generate neurons from iPSCs to study the mechanism(s) driving their maturation. The majority of the effort has focused so far on the transcriptional readout in terms of coding transcriptome. In this Research Topic, Kuruş et al. have extended the analysis to the non-coding transcriptome, focusing on long non-coding RNAs, transcripts generally longer than 200 nucleotides that do not generate any corresponding translated proteins and are emerging as crucial regulators of developmental and differentiation dynamics.

### Organoids as a potential alternative for basal progenitor research

In recent years, iPSCs- and ESCs-derived brain organoids have progressed to the point of being a viable alternative for the study of many aspects of neural development and evolution. While primary tissue remains the benchmark, its use presents many limitations and constraints, scientific, technical and ethical alike and is obviously not viable for extinct species. For all their advantages, model organisms, including ferret and even primates, by definition cannot give a full picture of the larger and more folded brains of humans and other hominids. Also, in-tissue genetic modification and labelling, e.g., *via* viral infection, electroporation and microinjection, can typically only reach subsets of cells. These limitations have greatly increased the interest in 3D alternatives, such as organoids. In this Research Topic, Kaluthantrige Don and Kalebic discuss advances in a crucial Frontier for brain organoid research, namely the recapitulation of basal radial glia (bRG) biology. In contrast to the apical ventricular zone, the complexities of the basal zones and their progenitors remain very challenging to replicate in organoids, constituting a major obstacle to the study of human brain evolution and modelling of the bRG-dependent human-specific neurodevelopmental disorders. Progress is being made, however, and the *in vitro* generation of proper subventricular zones, fiber layers and the neuronal layers may soon be on the horizon. These exciting challenges notwithstanding, the review also illustrates how organoids have already contributed to relevant discoveries regarding bRGs and its implications for the evolution of animals with higher encephalization.

## The genomic basis of recent human history

The availability of *in vitro* models recapitulating features of the developing human brain, the access to ancient genomes and the advent of genome editing motivated researchers to model (at least partially) features of ancient human brain in a dish. Of outstanding importance in fueling this line of interest is the insight and predictive power derived by comparing archaic genomes to the ones of contemporary humans. Buisan et al. provide us with the perspective from paleogenetics and ancient DNA by focusing on introgression deserts, which are parts of the genome showing no or very little traces of archaic DNA. By comparing publicly available RNA sequencing datasets, and by looking specifically at divergence in the transcriptional readout of desert regions, Buisan et al. show that the cerebellum, and other areas outside of the cortex exhibit the highest degrees of transcriptional divergence. In addition to this spatial and area-specific information, of note, time is also a crucial dimension when looking for differences; indeed, as the highest divergence was found in development. This observation is extremely relevant also in a physiopathological context, as many genes responsible for brain disorders (even the ones arising during adulthood) are most highly expressed during development, pointing to development as the most relevant window for human evolution and pathology alike.

## Concluding remarks

The temporal dissection summed up above clearly illustrates the potential derived from integrating different approaches and scales of analysis in the conjoined study of brain development and evolution. For it is at the spatiotemporal intersection, and along both the evolutionary and developmental axis, that the insights from the cutting edge experimental approaches and model systems discussed in this special issues, can most powerfully contribute to a contemporary molecular understanding of the human condition.

